# Pain-related fear in adolescents with chronic musculoskeletal pain: process evaluation of an interdisciplinary graded exposure program

**DOI:** 10.1186/s12913-020-5053-6

**Published:** 2020-03-14

**Authors:** C. Dekker, J. C. M. van Haastregt, J. A. M. C. F. Verbunt, J. R. de Jong, T. van Meulenbroek, H. F. M. Pernot, A. D. van Velzen, C. H. G. Bastiaenen, M. E. J. B. Goossens

**Affiliations:** 1 Department of Rehabilitation Medicine, Care and Public Health Research Institute (CAPHRI), Functioning and Rehabilitation, Maastricht University, Universiteitssingel 40, 6229 ET Maastricht, the Netherlands; 2 Department of Health Services Research, Care and Public Health Research Institute (CAPHRI), Maastricht University, Maastricht, the Netherlands; 3 Adelante, Center of Expertise in Rehabilitation and Audiology, Hoensbroek, the Netherlands; 4 Medicine, Laurentius Hospital Roermond, Roermond, the Netherlands; 5 Revant Rehabilitation center, Breda, the Netherlands; 6 Department of Epidemiology, Care and Public Health Research Institute (CAPHRI), Functioning and Rehabilitation, Maastricht University, Maastricht, the Netherlands; 7 Department of Clinical Psychological Sciences, Experimental Psychopathology, Maastricht University, Maastricht, the Netherlands

**Keywords:** Graded exposure in vivo, Adolescent, Chronic pain, Treatment fidelity, Feasibility

## Abstract

**Background:**

For studying the effectiveness of treatment, it is important to check whether a new treatment is performed as originally described in the study-protocol.

**Objectives:**

To evaluate whether an interdisciplinary graded exposure program, for adolescents with chronic musculoskeletal pain reporting pain-related fear, was performed according to protocol, and whether it is feasible to implement the program in rehabilitation care.

**Methods:**

A process evaluation where quantitative and qualitative data on participant characteristics (adolescents, parents and therapists), attendance and participants’ opinion on the program were collected, by means of registration forms, questionnaires and group interviews. To evaluate treatment fidelity, audio and video recordings of program sessions were analyzed.

**Results:**

Thirty adolescents were offered the program, of which 23 started the program. Adolescents attended on average 90% of the sessions. At least one parent per adolescent participated in the program. Analysis of 20 randomly selected recordings of treatment sessions revealed that treatment fidelity was high, since 81% of essential treatment elements were offered to the adolescents. The program was considered client-centered by adolescents and family-centered by parents. Treatment teams wished to continue offering the program in their center.

**Conclusion:**

The interdisciplinary graded exposure program was performed largely according to protocol, and therapists, adolescents and their parents had a favorable opinion on the program. Implementation of the program in rehabilitation care is considered feasible.

**Trial registration:**

Clinicaltrials.gov ID: NCT02181725 (7 February 2014).

## Background

Chronic pain in children and adolescents can substantially impact their health-related quality of life [1]. The pain can interfere with physical, psychological and social functioning and can cause serious psychological distress [1, 2]. In the long term, there is a risk that complaints continue to exist in adulthood [3–5]. Because of this wide impact, chronic pain in children and adolescent is considered a complex health problem that requires interdisciplinary treatment in case of a high level of pain-related disability [6–8]. Furthermore, in adolescents with chronic musculoskeletal pain with a high level of disability, there is an elevated prevalence of joint hypermobility [9–11], for which multidisciplinary treatment is advised [12].

It has been demonstrated that pain-related fear is an important factor in explaining the severity of pain complaints and pain-related disability in adolescents [13, 14]. When pain is perceived as a threat (e.g. as a sign of injury) this can provoke anticipatory fear responses, catastrophizing thoughts and associated protective and avoidant-behavior. Prolongation of these behaviors may have the paradoxical effect that fear, associated pain sensitivity and disability sustain [15]. For the adolescent population, this mechanism is explained in the interpersonal fear avoidance model of pain [16], that incorporates the important interaction between adolescent and parents, in addition to the original fear avoidance model of Vlaeyen and Linton [17]. Recently, exposure in vivo interventions showed promising results in reducing functional disability in various chronic pain conditions in adults [18–20].

Because of demonstrated effectiveness in adults and supporting evidence for the applicability of the underlying theoretical model in adolescents, an interdisciplinary graded exposure program was developed to improve adolescent functional disability by reducing pain-related fear and catastrophizing of both the adolescent and the parents. This is accomplished by challenging catastrophizing thoughts and performing feared and avoided activities in order to improve physical, psychological and social functioning. To our knowledge, graded exposure in vivo has not been studied for its effectiveness and feasibility among adolescents before.

This paper describes a process evaluation according to the framework of Saunders and colleagues [21]. The objectives are to evaluate whether the exposure program was performed as intended and can be implemented in practice. The process evaluation was performed alongside a multicenter randomized controlled trial, in which the effectiveness of the newly developed exposure program compared to usual care was assessed [22]. As recommended by Oakley and colleagues [23], the process evaluation was performed before the results regarding the effectiveness of the exposure program were analyzed. The assessed factors are the extent to which the exposure program was delivered according to protocol (treatment fidelity and dose delivered), the extent to which the adolescents actively participated in the exposure program (dose received – exposure) and the opinion of patients, parents and treatment teams on the exposure program (dose received satisfaction and context).

## Methods

### Description of the intervention

**Fig. 1:**
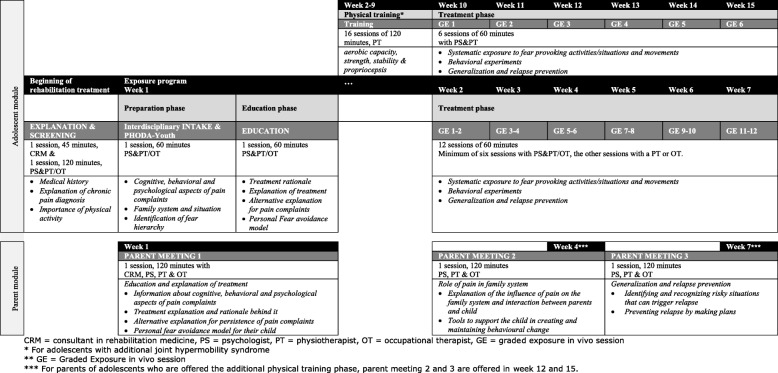
Planning of rehabilitation treatment with the interdisciplinary graded exposure program. CRM = consultant in rehabilitation medicine, PS = psychologist, PT = physiotherapist, OT = occupational therapist, GE = graded exposure in vivo session. * For adolescents with additional joint hypermobility syndrome. ** GE = Graded Exposure in vivo session. *** For parents of adolescents who are offered the additional physical training phase, parent meeting 2 and 3 are offered in week 12 and 15

The graded exposure program aims to restore the adolescents’ age appropriate level of functioning and decrease functional disability by activating and invalidating catastrophizing thoughts about feared and avoided movements or activities. The exposure program consists of an adolescent module, and a parent module that was offered in parallel with the adolescent module. For a subgroup of adolescents the adolescent module was combined with physical training if they were diagnosed with joint hypermobility syndrome. The modules of the program are described in Fig. 1.

The exposure program was offered by an interdisciplinary rehabilitation treatment team that is led by a consultant in rehabilitation medicine, and further consisted of a psychologist, and a physiotherapist or occupational therapist [24]. When an adolescent is referred to rehabilitation care, treatment started with an intake by the consultant in rehabilitation medicine and a screening. The first goal of the screening was determining which program (e.g. interdisciplinary rehabilitation program, or other) was most appropriate for a particular adolescent. During the intake and screening the medical history, the current medical condition and the expected capacity of the adolescent were evaluated. The physical and psychosocial factors related to functioning, including pain-related fear were assessed [22]. In addition, an explanation of the chronic pain syndrome was provided to the adolescent and parents. Dependent on the level of disability, the complexity of the pain problem, the level of pain-related fear, psychosocial problems, and the presence of joint hypermobility syndrome, the consultant then decided, supported by the results of the screening performed by the interdisciplinary team, whether the adolescent was eligible to participate in the study [22].

Once randomization indicated that an exposure program had to be started (and this will be the focus of this process evaluation), first an intake was performed by a psychologist together with a physiotherapist and/or occupational therapist. During this interdisciplinary intake, with the adolescent (and parent(s)), a biopsychosocial perspective on pain and the role of fear were further explained and were placed within the context of the family system. The Photograph Series of Daily Activities – Youth (PHODA-Youth) was used to systematically build a personalized hierarchy of activities that were feared and avoided by the adolescent [25]. Subsequently, the treatment rationale was explained in an education session, and the fear avoidance model was personalized using the life story of the adolescent. Thereafter, the twelve exposure sessions took place, in which the adolescent was systematically exposed to feared and avoided activities during behavioral experiments, based on the personalized hierarchy. The behavioral experiments were performed to challenge catastrophizing thoughts and execute activities in order to decrease pain-related fear [17].

Adolescents with additional joint hypermobility syndrome received physical training prior to the exposure sessions, to improve their physical fitness and muscle strength [26, 27] (Fig. 1). This physical training was offered by the physical therapist, in a gym and a swimming pool. The training was part of the exposure program to ensure that physical complaints of the joint hypermobility syndrome would not hinder the execution of the feared activities.

The parent module consisted of three meetings where parents were educated on the treatment rationale, the interpersonal fear avoidance model, the role of pain within the family system, and the parents’ role in providing support and preventing relapse (Fig. 1).

### Study design

This process evaluation used a multimethod approach including prospective and retrospective qualitative and quantitative components. Data were collected between February 2014 and February 2017, before the start of the exposure program, during the program and after the program had finished. Ethical approval for this study was provided by the Medical Ethics Committee Academic Hospital Maastricht/Maastricht University, the Netherlands, NL47323.068.13.

### Population

The population who participated in this process evaluation consisted of the adolescents and their parents who attended the program, and the treatment teams who offered the program. Adolescents, 12–21 years old, were eligible for participation in the study if they were referred to outpatient rehabilitation treatment for chronic musculoskeletal pain in one of the four participating rehabilitation centers in the Netherlands (one of the centers was the department of rehabilitation medicine of a general hospital). Adolescents were excluded if there was a suspicion of a medical (orthopedic, rheumatic or neurological) disease that could fully explain the current level of severity of pain complaints, if there was a suspicion of an underlying psychiatric disease that would hamper rehabilitation treatment, or in case the adolescent was pregnant.

### Training of the treatment teams

The treatment teams of three of the four participating treatment centers received a 4-day training in the exposure program and study procedures before the start of the intervention period. In one center the team was already trained in the exposure program and worked with the program protocol for 2 years already.

Training was conducted by experienced behavioral therapists and experts in graded exposure in vivo treatment, and members of the study team. During the training, the program modules and study procedures were explained and practiced by the treatment teams by means of role plays. During the study period, supervision was offered through telephone or email contact with the trainers. Treatment teams received a detailed treatment manual (protocol) with instructions for each program session.

### Data collection

The description of the data collection is in chronological order. Table 1 shows the components of the process evaluation, the operationalization of the components, and the data collection methods. The training of the treatment teams was evaluated by means of a questionnaire (TE), on the last training day. The questionnaire consisted of closed-end questions that could be answered on a 10-point scale ranging from 1 (totally disagree) to 10 (totally agree). Adolescents and parents received a questionnaire on participant characteristics and treatment expectancy (Q1), in the week before the start of the exposure program. An adapted version of the Credibility/Expectancy questionnaire was used to measure treatment expectancy and rationale credibility in adolescents who were about to start the exposure program [28, 29]. Formulation of the questions was adapted to correspond with the treatment goals in the program protocol. The adapted Credibility/Expectancy questionnaire consisted of 8 questions, rated on a 9-point Likert scale ranging from 1 (not at all) to 9 (very much). Questions concern the credibility of the rationale for the treatment and treatment goals, and the improvements adolescents expect to make concerning these treatment goals. Higher scores indicate a higher credibility and higher expectations.

**Table 1:** Components of the process evaluation and data collection methods

	TE	Q1	AV	RF	Q2	GI
**Performance according to protocol** (Treatment fidelity and dose delivered)						
Delivery of 8 essential elements of preparatory phase *(Elements are specified in* Table 2*)*			x			
Delivery of 15 essential elements of education phase *(Elements are specified in* Table 2*)*			x			
Delivery of 11 essential elements of treatment phase *(Elements are specified in* Table 2*)*			x			
Planning of the treatment sessions according to protocol				x		x
**Adolescent participation in the program** (Dose received – exposure)						
Extent to which adolescents attended the treatment sessions				x		x
**Opinion on the program** (Dose received-satisfaction)						
Opinion of the adolescents		x			x	
Opinion of the parents		x			x	
Opinion of the treatment teams						x
Training	x					

*TE* Training evaluation questionnaire, *Q1* Questionnaire on characteristics of study population, *AV* Audio and video recordings, *RF* Registration forms, *Q2* Questionnaire on the opinion on the program; Giving Youth A Voice Questionnaire for the adolescents and Measure of Processes of Care Questionnaire for the parents, *GI* Group interview with treatment teams

Performance according to protocol is an integrated measure of treatment fidelity (i.e. a qualitative indicator that refers to the extent to which the essential treatment elements were offered in a qualitative good way to the adolescents), and dose delivered (i.e. a quantitative indicator that refers to the extent to which all treatment elements were offered in the prescribed (minimum) dose to the adolescents, and protocol deviations). Treatment fidelity was assessed for the adolescent module and was expressed as the percentage of essential treatment elements executed by the treatment teams. To enable analysis, the adolescent module was divided into phases: a preparation phase, which included the interdisciplinary intake and PHODA-youth, an education phase, which included the education session, and a treatment phase, which included the graded exposure in vivo sessions. Audio and video recordings (AV) were made by the therapists, during the three phases of the program. Specifically, the intake, education and graded exposure in vivo treatment sessions of the adolescent module were recorded. Physical training, explanation on the chronic pain syndrome, screening, and parent meetings were not recorded. Information about attendance of adolescents and parents, and protocol deviations by the treatment teams was noted on registration forms (RF; a form to be filled in by a professional, with specific questions concerning the process of care delivery of one specific patient).

The adolescents’ opinion on the treatment they received during the program was measured with the Giving Youth A Voice questionnaire and for the parents’ opinion, the Measure of Processes of Care questionnaire was used [30–33]. Adolescents and their parents were asked to complete these validated questionnaires at the end of the exposure program (Q2). Both the Giving Youth a Voice questionnaire and the Measure of Process of Care questionnaire measure client-centeredness and family-centeredness of rehabilitation services respectively. Both questionnaires consist of 20 items that can be scored on a scale of 1–7, with 7 meaning a specific action or behavior of the therapist has occurred very often, to 1 meaning this specific action or behavior has never occurred. Mean subscale scores for both the adolescent and parent questionnaire were computed. Higher ratings reflect a more favorable opinion of client- or family centeredness with the program they received. Scores were dichotomized (lowest four values in one category and highest three values in another category) to identify the lowest scoring items to indicate possibilities for program improvements.

A group interview (GI) was planned in each of the four treatment centers after all participants completed their exposure programs. For each meeting, the complete treatment team that offered the exposure program was invited. A topic list with predetermined open-ended questions was used as interview guide. The interview was conducted by two members of the study team, and audio recorded after consent. The evaluation focused on the performance of the program according to protocol, reasons for protocol deviations (including context factors), attendance and participation of the adolescents during the program, and opinion of the therapists on the exposure program protocol they had been working with.

### Data analysis

The quantitative data from the questionnaires and registration forms were analyzed using descriptive statistics in IBM SPSS Statistics for Windows, version 25 (IBM Corp, Armonk, NY, U.S.A.).

Treatment fidelity of the adolescent module was analyzed with an adapted version of the Method of Assessing Treatment Delivery, described by Leeuw and colleagues [34]. Five steps were taken. In step 1, therapists were asked to (audio or video) record sessions in three different phases of the program. After the intervention period, the audio and video recordings were collected by the study team, randomly numbered, sorted per treatment center and categorized in the three program phases. For each treatment center, four sessions were randomly selected for analysis, at least one from each phase. Since one treatment center treated as much adolescents as the other three centers together, eight recordings were selected from this center. This resulted in 20 recordings that were selected for analysis. In step 2 and 3, two independent raters scored whether pre-specified treatment elements were present or absent (Table 2). The raters were a master student in developmental psychology and a health scientist. They received 1 day training in the treatment protocol and scoring of the three treatment phases. In step 4, inter-rater reliability between the two raters was assessed by calculating Cohen’s kappa for the three phases together. In step 5 protocol adherence (treatment fidelity) was calculated. The treatment elements in the scoring forms have been scored present or absent, but these were categorized in Essential-and-Unique (EU), Essential-but-not-unique (E), Unique-but-not-essential (U), Compatible, but not essential and not unique (C) and Prohibited (P) elements for the program [34]. To calculate protocol adherence, the percentage essential (EU and E) elements was calculated for each phase and for all three phases together, and should exceed 70% for sufficient protocol adherence [34]. This percentage was calculated by dividing the number of observed essential elements by the maximum possible number of these elements.

**Table 2:** Percentage of occurrences observed of the Essential^a^ elements of the of the exposure program

No.	Specific element	Category	Percentage^b^
**PREPARATION PHASE** – *n* = 8 sessions evaluated			
P2	There is good teamwork between the therapists and adolescent	E	100
P3	The adolescent’s concern/fear with regard to activities is being discussed	EU	100
P6	The adolescent assesses the level of perceived threat value of daily activities (PHODA)	EU	94
P8	The aim of the treatment session is explained to the adolescent	E	88
P9	The therapists respond understandingly to the problems expressed by the adolescent	E	100
P10	Photographs of daily activities are being used	EU	88
P14	A hierarchy is being developed based on the threat value of daily activities (PHODA)	EU	88
P17	It is discussed how parents react to the disability and pain of their child.	E	13
	***Mean proportion of essential preparation phase elements (%)***		***84***
**EDUCATION PHASE** – *n* = 5 sessions evaluated			
E1	It is emphasized that in chronic pain no clear relationship exists between pain and injury	E	100
E3	The adolescent’s concern/fear with regard to activities is being discussed	EU	100
E4	There is good teamwork between the therapists and adolescent	E	100
E5	The aim of the treatment session is explained to the adolescent	E	80
E9	A biomechanical approach to pain is being discouraged	E	70
E11	It is emphasized that pain reduction is not a therapy goal	E	70
E12	The adolescent is being actively involved in the explanation of the therapy	E	100
E13	It is explained that the treatment is aimed at verifying examining cognitions	EU	70
E14	A bio-psycho-social approach to pain is being explained	E	100
E15	It is emphasized that all activities are possible	E	30
E17	The drawbacks of inactivity are being explained	E	70
E21	The circular model pain-pain cognitions- avoidance – pain is being explained	EU	90
E22	The therapists respond understandingly to the problems expressed by the adolescent	E	90
E23	It is explained that the aim of the therapy is an increase in activity level	E	40
E25	The motivation of the parents for the treatment of their child is being checked	E	10
	***Mean proportion of essential education phase elements (%)***		***79***
**TREATMENT PAHSE** – *n* = 7 sessions evaluated			
T1	Homework is being assigned	E	64
T3	The adolescent’s concern/fear with regard to activities is being discussed	EU	100
T4	The aim of the treatment session is explained to the adolescent	E	86
T5	A catastrophizing cognition is being identified	EU	100
T6	There is good teamwork between the therapists and adolescent	E	100
T8	A behavioral experiment is being performed	EU	100
T9	The therapists respond understandingly to the problems expressed by the adolescent	E	93
T11	Activities from the hierarchy or based on threat value are being performed	EU	93
T14	Clear agreements are made about the way in which activities should be carried out (e.g. how often, how high the jumps should be, how to bend down)	EU	50
T15	Homework is being evaluated	E	44
T21	It is discussed how parents react to the activities that the adolescent has started to perform.	E	29
	***Mean proportion of essential treatment phase elements (%)***		***79***

^a^Essential treatment elements are both Essential-and-Unique (EU) and Essential-but-not-unique (E) treatment elements (Method of Assessing Treatment Delivery, described by Leeuw and colleagues [34]*)*

^b^The reported percentage reflects the rating of both raters, therefore for each session evaluated, two ratings were used to calculate the percentage

The group interviews were recorded and transcribed. Analysis entailed open, axial and selective coding with the aim of proving an exploratory description of the treatment teams experiences with the program and their opinion about the program.

## Results

### Response and characteristics of the study population

Between August 2014 and December 2016 30 adolescents were offered the exposure program, of which process data was available for 23 adolescents. (for more information, a trial flow chart related to the effect evaluation is available as supplementary File 1). For six of the 23 adolescents, parents did not complete any questionnaires because they did not consent to participate in the study (*n* = 5), or the adolescent was 18 years and decided to participate without parents (*n* = 1). Therefore, questionnaire data was available for 31 parents (14 parent couples, twice only a mother, once only a father). The mean age of the parents was 48 years (*SD* = 5.82, range 33–64 years). Seven out of the 30 adolescents (24%) and their parents did not start the program, because the complaint resolved (*n* = 2), the situation deteriorated so that outpatient treatment was no longer indicated (*n* = 1), adolescent refrained from rehabilitation treatment before start of the program (*n* = 1) or loss of contact (*n* = 2). In addition, one adolescent started after the study period. Of the 23 adolescents (76% out of 30 adolescents) for whom process data was available, 18 adolescents received the standard three-phase adolescent module. The remaining five adolescents received the adolescent module with additional physical training.

The mean age of the adolescents analyzed *(n* = 23) was 15.6 years (*SD* = 1.7, range 12–18 years). Twenty-two adolescents were female (96%). Almost half of the adolescents (48%, *n* = 10) had a lower education, being elementary school or lower vocational education (*n* = 2 missing). For the majority of adolescents (80%) their pain complaints started more than 1 year ago (*n* = 3 missing). Twelve adolescents (60%, *n* = 3 missing) have a relative with pain complaints.

The participating members of the four interdisciplinary treatment teams (*n* = 20) were 4 consultants in rehabilitation medicine, 7 psychologists, 5 physiotherapists and 4 occupational therapists. For the training in the exposure program, thirteen of the sixteen (81%) members of the treatment teams participated (one team of four members was already trained). After the intervention period, 14 (70%) of the 20 invited therapists were present at the group interviews. Here, one physician in rehabilitation medicine, four psychologists and one occupational therapist were unable to attend.

### Evaluation of the training of the treatment teams

Evaluation of the training revealed that the participants overall had a favorable opinion on the training. They considered the training relevant, the trainers competent, and they indicated that the training formed a good preparation for conducting the exposure program. However, they also made some recommendations for improving the training and preparation for the treatment. In addition to the training, they would like individual feedback on their performance with real patients to increase confidence. Therefore, they were offered the possibility to consult the trainers before and during the intervention period. However, this possibility was rarely used. Furthermore, the team members highlighted the importance of the whole team, including the consultant in rehabilitation medicine, to participate in the training (two consultants and one psychologist were unable to attend all training days, due to different circumstances). In addition, it was recommended that training should immediately be followed by the opportunity to work with adolescents in practice so that treatment teams could develop their skills. Due to practical reasons, such as the duration of the procedure for ethical approval of the study, and the set-up of the intervention and study procedures in the treatment centers, this was not immediately possible.

### Performance of the program according to protocol (treatment Fidelity and dose delivered)

In all centers the adolescent and parent module were offered as intended. Two centers did not offer the physical training, because no adolescents fulfilled the criteria for additional joint hypermobility syndrome. Some protocol deviations were made in all centers due to practical reasons, such as illness of a team member or absence of a swimming pool for training. *(F.e:* In absence of a swimming pool for training in one center, the elements of the training sessions in the swimming pool were all conducted on land (with comparable impact) by using a treadmill or exercise bike. If a therapist was unable to provide the exposure sessions due to illness, the sessions were performed by another therapist of the individual treatment team.

Also, 12 parent modules were offered in a group setting and 11 modules were offered to individual parents/parent couples. Reason to offer the parent module individually was due to a low number of included adolescents in two centers.

To analyze treatment fidelity, 105 of total 266 treatment sessions of the adolescent module (39%) were recorded. According to the therapists, the main reason for not recording sessions was that it was forgotten; either to bring the recorder to the session or to turn it on. A random selection of 20 recordings (preparation phase *n* = 8, education phase *n* = 5, treatment phase *n* = 7, in total 16 audio and four video recordings) was scored by two independent raters. Interrater reliability between the two raters was Cohen’s kappa 0.72 for all three phases together. Disagreement between the raters was not resolved. The reported percentages in Table 2 are calculated with the ratings of the two raters.

Table 2 shows the percentages of observed essential protocol elements in all three phases of the adolescent module. Treatment fidelity, scored as the mean proportion of essential treatment elements that have been offered to the adolescents, is 84% for the preparation phase, 79% for the education phase and 79% for the treatment phase. For the three phases together, treatment fidelity was 81%.

Some elements concerning parent involvement (P17, E25 and T21) score below 30%, indicating that these elements were seldom performed during the program sessions of the adolescent. Further, elements on the aim of the program (E15 and E23), and agreements and homework (T1, T14 and T15) scored relatively low, indicating that the aim of the treatment was not clearly communicated to the adolescent during the education phase and that agreements and homework were not clearly communicated during the treatment phase.

### Active participation in the program (dose received exposure)

Adolescents in the standard module (*n* = 18) attended on average 12.7 of the 14 program sessions (90%, range 8–14 sessions). When including the 7 adolescents who did not start the program, and therefore attended 0 program sessions of the standard module, this percentage drops to 65%, meaning an average attendance of 9,1 sessions of the 14 program sessions potentially offered. Adolescents who received the standard module including physical training (*n* = 5) attended on average 21.6 of the 24 planned program sessions (90%, range 20–24 sessions). In the latter group 14.8 of the 16 (93%, range 12–16) of the physical training sessions and 6.8 of the 8 (85%, range 4–8) of the graded exposure sessions were attended. Of the 23 adolescents who started the program, 21 adolescents completed their program. Two adolescents did not complete their program because they changed treatment (*n* = 2). These two adolescents attended 8 of the 14 (57%) and 20 of the 24 (83%) planned sessions respectively.

For the parent module, attendance was retrospectively assessed in the group interview for the parents of all 23 adolescents. For all 23 adolescents, at least 1 parent attended the parent module. Approximately half of the modules were attended by both parents, the other half by one parent (for six parent couples it could not be retrieved whether one of both parents attended).

### Opinion of adolescents, parents and treatment teams on the program

The adolescents’ mean credibility score for the treatment rationale before the start of the exposure program was 17.7 (*SD =* 5.1, on a scale of 3–27) and mean expectancy was 13.2 (*SD =* 2.6, on a scale of 2–18), scored by 20 adolescents (*n* = 3 missing). These scores indicate that adolescents assign relatively moderate credibility that the exposure program will increase their functioning and participation in social activities despite their pain. Furthermore, these scores indicate the adolescents have relatively high expectations with regard to the improvements the exposure program can cause in functioning and participation in social activities.

The Giving Youth a Voice questionnaire was completed by 17 adolescents (which is 74% of adolescents who started the program, *n* = 5 questionnaires missing; which is 57% of all adolescents randomized to the program, *n =* 13 questionnaires missing), reflecting their opinion on the exposure program. Mean scores on the subscales are presented in Table 3. Overall, the care these adolescents have received was scored as highly client-centered, since scores on all subscales are close to the maximum score of 7. The items ‘How much do the people who work with you inform you of how treatments might harm you or help you?’ and ‘How much do the people who work with you offer you useful information about how you are doing?’ were scored by 24% of the adolescents in the lowest category (the lowest 4 answering scores). This is where the behavior of the therapists had the lowest scores on client-centeredness.

**Table 3:** Opinion about the client- and family centeredness of the program

Adolescents’ opinion		Parents’ opinion	
Giving Youth A Voice Questionnaire-20 subscales (n items)		Measure of Processes of Care Questionnaire-20 subscales (n items)	
	Mean^a^ (sd)		Mean^a^ (sd)
Supportive and Respectful Relationships (5)	5.8 (1.66)	Enabling and Partnership (3)	4.7 (1.63)
Information Sharing/Communication (5)	6.1 (1.29)	Providing General Information (5)	4.5 (1.61)
Supporting Independence (5)	6.0 (1.22)	Providing specific information about the child (3)	5.3 (1.35)
Teen Centered Services (5)	6.2 (1.09)	Coordinated and comprehensive care for child and family (4)	5.7 (1.14)
		Respectful and supportive care (5)	4.9 (1.31)

^a^Mean scores on a scale 1–7, with higher scores reflecting a more favorable opinion on the behaviors of the therapists reflected in the subscales. The mean values of the subscales of the GYV-20 were calculated from 17 questionnaires completed by the adolescents. The mean values of the subscales of the MPOC-20 were calculated from 31 questionnaires completed by the parents

The Measure of Processes of Care questionnaire was completed by all participating parents (*n* = 31). Mean scores on the subscales are presented in Table 3. Overall, the parents scored the care they received for their adolescent as family-centered, since mean scores on the subscales are on the higher end of the answering scale (range 1-7). In 8 of the 20 questions, more than 75% of the parents scored therapist behaviors to occur at least more than sometimes (highest category, highest three answering scores). The items the parents scored most in the lowest answering category (57 and 55%) were ‘To what extent do the people who work with your child treat you as an individual rather than as a ‘typical’ parent of a child with a disability?’, and ‘To what extent do the people who work with your child have information available to you in various forms, such as a booklet, kit, video, etc.?’ For these behaviors of the therapist the parents’ ratings were the lowest on family-centeredness.

All participating treatment teams had a positive opinion about the exposure program, although most therapists perceived practice of the program challenging. The teams, however, intend to continue the use of the exposure program within their rehabilitation center, by integrating the program in their regular care. Multiple reasons were mentioned for the willingness to implement the exposure program. The exposure program was found to be suitable specifically for adolescents with chronic musculoskeletal pain reporting pain-related fear and avoidance behavior. According to the opinion of the treatment teams, the program could cause behavioral change in activity performance in a relatively short time frame. Furthermore, the program provided a clear outline for the adolescents in terms of planning and working towards their treatment goals, which worked well with this patient group. The parent involvement was much appreciated and considered essential to preserve treatment gains at home. However, due to the relative short duration of the intervention and the experienced relatively narrow focus of the intervention (improve functional disability by targeting pain-related fear) concerns were expressed regarding the long-term effects of the program. Especially with regard to relapse into previous pain behavior, and treatment of (psychological) co-morbidity, treatment teams expressed their doubts regarding treatment effectiveness. Moreover, guidance on how to handle in case of relapse in previous pain behavior was considered to be too restricted in the program protocol.

The psychologists and physiotherapists/occupational therapists also indicated they enjoyed and preferred working in a duo during the program sessions. They could support each other during program sessions; it enabled evaluation of the sessions (they felt better informed of what happened in the treatment because they were there), and it improved communication with adolescents and parents.

## Discussion

This process evaluation assessed whether a graded exposure program offered in interdisciplinary rehabilitation care was performed according to protocol and whether it would be feasible to implement the program in rehabilitation care. Data for the process evaluation was available for 76% of the adolescents who completed the program. Treatment fidelity was high in all three treatment phases, since on average 81% of the essential treatment elements were offered to the adolescents. Adolescent participation was high, with attendance rates of 90% and with at least one parent participating in the parent module. Adolescents and parents considered the exposure program client-centered and family-centered respectively. Adolescents considered the program moderately credible and had relatively high expectations about attaining the treatment goals (improved functioning and participation in social activities despite pain) after their first visit to the consultant in rehabilitation medicine. Furthermore, treatment teams had a favorable opinion about the content of the exposure program, and had the intention to implement the program in regular care in their rehabilitation center. Psychologists, physiotherapists and occupational therapists appreciated working jointly together in duo’s in the exposure sessions.

It is difficult to compare these results with other process evaluations of rehabilitation treatment for adolescents with chronic pain, as no studies of this kind exist to our knowledge. Leeuw and colleagues [34] provided an illustration of the application of their Method of Assessing Treatment Delivery in a rehabilitation treatment in adults with chronic low back pain. Although the aims of the studies were slightly different (Leeuw aimed to evaluate whether treatment comparisons were fair, our aims was to evaluate whether the exposure program was performed according to protocol) similar checklists to score treatment elements were used. Contrary to the study by Leeuw et al., where two interventions were compared, analysis focused in our study only on the performance of essential treatment elements in one intervention condition. Although the majority of essential elements were offered to the adolescents, this study showed that there were some elements that were not offered as planned. Elements concerning explanation of the aim of the treatment, elements concerning agreements and homework, and elements concerning parent involvement, were scored only few times. Reasons for this are unknown. However, as these elements are considered to be essential and therefore important for the high quality delivery of the intervention, performance might be enhanced by stressing these topics in the program protocol and during the training of the treatment teams.

This study has some strengths and limitations that need to be mentioned. Most important limitations concern the small sample size of this study and the problems with missing data. At the onset of the research project, four treatment centers were invited to participate in the project in order to reach the desired treatment capacity for a properly powered RCT. However, the number of adolescents that was included in the RCT did not reach the calculated sample size. A subgroup of adolescents identified as potential research participants declined participation in a scientific study due to various reasons, often related to extra efforts as compared to normal treatment outside the RCT. These patients did, however, follow (regular) treatment. As a result, the potential number of patients for whom the program may be a treatment option is higher than the actual number of patients that received the program within this study.

Concerns may be raised about the relatively large proportion of missing questionnaire data presented in this manuscript. With the high drop out rates in this study, it has to be acknowledged that there is the possibility of differential drop out and especially with the small study population, small systematic differences between participants and drop outs can have a significant influence on the evaluation of the client-centeredness of the treatment program. In the total RCT, missingness of the data was assumed to be random since missingness could not be related to any measured outcome or other measured variable (such as age or education). Furthermore, reasons for not participating in the study were various and did not lead to the suspicion of volunteer bias.

Concerning the data collected at the parent level, another limitation needs attention. Although data of two parents of the same child is correlated, this data was treated as independent in this study. Treating the questionnaire data of the parents as independent probably (mildly) inflated the high satisfaction rates of the treatment. Another limitation is the possibility that socially desirable answers were given by the adolescents, parents and treatment teams, when asked about the exposure program. To limit this risk, adolescents and parents received all an individual link to their questionnaires by email, which they could complete at home and results would only be published at group level. Furthermore, the group interviews with the treatment teams were performed by two members of the study team. The risk of obtaining socially desirable answers might have been lower if these meetings were led by independent interviewers, not involved in the study. The different opinions of the treatment teams about the program were derived from group interviews. Because data from group interviews was used, no quantitative data on the frequencies of the reported opinions on an individual level is available. The results on the treatment teams opinions should only be interpreted as an exploration of how treatment teams feel about offering the program.

Moreover, treatment teams were aware that the recordings they made of their treatment sessions would be used to evaluate their performance according to protocol, which may have influenced their behavior. This raises another limitation. Only 39% of the treatment sessions were recorded which may have biased the available data. It might give the idea that treatment teams who were less experienced were afraid to record their treatment sessions.

An important strength of this study is the analysis of treatment fidelity with the adapted Method of Assessing Treatment Delivery. Applying this method, allowed real insight into the degree to which essential protocol elements were applied by the members of the treatment teams. Together with the attendance registration, both the quantity (dose delivered), and the quality (treatment fidelity) of the adolescent module were assessed. A limitation to this approach in our study was that treatment fidelity was not evaluated for the parent module. The parent module was intended to be a group intervention and it was possible that parents who did not consent to participate in the study would be present. Therefore, it was considered not possible to record these parent meetings. Similar, treatment fidelity was not assessed for the physical training of the adolescents, so we lack insight in the fidelity of this part of the program. By all means, evaluation of treatment fidelity of all parts of the exposure program would have provided a more complete picture of the quality of delivery of the program. Furthermore, as in the approach of Leeuw and colleagues [34], our evaluation also focused on the mere assessment of treatment delivery. Treatment receipt by the patient, and enactment of the patient upon treatment were disregarded, two components that by some authors have previously been described as part of treatment fidelity as well [35, 36].

Compared to similar graded exposure interventions in adults with chronic pain, in which a psychologist is present at each exposure session, the psychologist was allowed to be present at least every second exposure session in the treatment phase in this study. Since the members of the treatment teams expressed several advantages of working as a duo, we recommend to abide by this minimum of duo sessions when implementing the program into rehabilitation care. Further, although treatment teams were trained for 4 days, they perceived good practice of the program challenging. Hence, teams indicated they needed to gain real world experience and wished for feedback on their individual functioning. The possibility to contact the trainers during the intervention was rarely used. In the future, the organization of regular intervision-sessions within the centers can help to overcome this. To ensure high quality program delivery, de Jong and Verbunt [37] also emphasize the importance of continuous training, real world practice and regular team intervision meetings. Therefore, the 4 days of training is recommended to be a minimum and it is recommended to incorporate regular intervision-meetings and yearly refresher courses when working with the program.

## Conclusion

Based on the results of this process evaluation, we conclude that the interdisciplinary exposure program has been performed largely according to protocol and that treatment fidelity of the adolescent module was high. In addition, the participants in general actively participated in the adolescent module. The delivery of the exposure program was perceived client-centered by the adolescents and family-centered by the parents. Treatment teams expressed a favorable opinion about the exposure program and intend to implement the program within their rehabilitation centers. Therefore, implementation of the program is considered feasible in specialized rehabilitation care.

In case the effect evaluation of the exposure program reveals positive results on treatment effectiveness, implementation of the program in specialized rehabilitation care is recommended. However, for future research, investigation of treatment fidelity (including treatment receipt and participant enactment) of the currently not evaluated parent module and physical training is recommended, since analyzing both the complete adolescent module and parent module will provide the most complete picture of treatment fidelity of the program.

## Supplementary information

**Additional file 1 Figure S1** Adolescent flow through the RCT.

## Data Availability

The datasets used and/or analysed during the current study are available from the corresponding author on reasonable request.

## Abbreviations

AV: Audio and video recordings
C: Treatment elements: Compatible, but not essential and not unique
E: Treatment elements: Essential-but-not-unique
EU: Treatment elements: Essential-and-Unique
GI: Group interview
P: Treatment elements: Prohibited
PHODA- Youth: The Photograph Series of Daily Activities – Youth
RF: Registration forms
U: Treatment elements: Unique-but-not-essential
