# Supplementary material for: Pain-related fear in adolescents with chronic musculoskeletal pain: process evaluation of an interdisciplinary graded exposure program
Source: BMC Health Serv Res. 2020 Mar 14;20:213. doi: 10.1186/s12913-020-5053-6 (PMC7071667; doi:10.1186/s12913-020-5053-6)
Supplement: Supplementary file 1 — Additional file 1 Figure S1 Adolescent flow through the RCT. [file 12913_2020_5053_MOESM1_ESM.docx]

Declined to participate (*n* = 17)

- Unwilling to participate in study (*n* = 4)
- Too busy with school (*n* = 3)
- Unwilling to be randomized (*n* = 2)
- Traveling distance to the center is too long (*n* = 2)
- Did not fulfill eligibility criteria after all (*n* = 1)
- Not open for alternation of treatment policy (*n* = 1)
- Was referred to a treatment center outside the study (*n* = 1)
- Participation places too much emphasis on the complaints (*n* = 1)
- Reason unknown (*n* = 2)

Invited to participate after eligibility assessment (*n* = 77)

Randomized (*n* = 60)

CARE AS USUAL (*n* = 30)

Allocated to the intervention (*n* = 30)

- Received allocated intervention (*n* = 27)
- Did not receive allocated intervention (*n* = 3)
- Complaint resolved before start of intervention (*n* = 1)
- Refrained from rehabilitation treatment (*n* = 1)
- Unknown (*n* = 1)

EXPOSURE PROGRAM (*n* = 30)

Allocated to the intervention (*n* = 30)

- Received allocated intervention (*n* = 23)
- Did not receive allocated intervention (*n* = 7)
- Refrained from rehabilitation treatment (*n* = 1)
- Complaint resolved before start of intervention (*n* = 1)
- Situation deteriorated (*n* = 1)
- Complaint resolved before start of intervention (*n* = 1)
- Loss of contact (*n* = 2)
- Treatment started after end of study (*n* = 1)

Discontinued intervention (*n* = 2)

- Switched to inpatient treatment (*n* = 1)
- No specific treatment goals (*n* = 1)

Discontinued intervention (*n* = 2)

- Change of treatment (*n* = 2)

Analyzed (*n* = 28)

Adolescents completely missing at baseline (*n* = 2)

Excluded from analysis (*n* = 0)

Measurements

- 2 months - *n* = 24 (86%)
- 4 months - *n* = 19 (68%)
- 10 months - *n* = 15 (54%)
- 12 months - *n* = 12 (43%)

Analyzed (*n* = 25)

Adolescents completely missing at baseline (*n* = 5)

Excluded from analysis (*n* = 0)

Measurements

- 2 months - *n* = 19 (76%)
- 4 months - *n* = 18 (72%)
- 10 months - *n* = 7 (28%)
- 12 months - *n* = 10 (40%)

**Figure 1** Adolescent flow through the RCT
